# Physical partisan proximity outweighs online ties in predicting US voting outcomes

**DOI:** 10.1093/pnasnexus/pgaf308

**Published:** 2025-10-21

**Authors:** Marco Tonin, Bruno Lepri, Michele Tizzoni

**Affiliations:** Department of Sociology and Social Research, University of Trento, Via Verdi 26, Trento I-38122, Italy; Mobile and Social Computing Lab (MobS), Fondazione Bruno Kessler (FBK), Via Sommarive 18, Trento I-38123, Italy; Mobile and Social Computing Lab (MobS), Fondazione Bruno Kessler (FBK), Via Sommarive 18, Trento I-38123, Italy; Department of Sociology and Social Research, University of Trento, Via Verdi 26, Trento I-38122, Italy

**Keywords:** co-location, vote choice, social networks, partisan segregation, political polarization

## Abstract

Affective polarization and increasing social divisions affect social mixing and the spread of information across online and physical spaces, reinforcing social and electoral cleavages and influencing political outcomes. Here, using individual survey data and aggregated and de-identified co-location and online network data, we investigate the relationship between partisan exposure and vote choice in the United States by comparing offline and online dimensions of partisan exposure. By leveraging various statistical modeling approaches, we consistently find that partisan exposure in the physical space, as captured by co-location patterns, more accurately predicts electoral outcomes in US counties, outperforming online and residential exposures. Similarly, offline ties at the individual level better predict vote choice compared to online connections. We also estimate county-level experienced partisan segregation and examine its relationship with individuals’ demographic and socioeconomic characteristics. Focusing on metropolitan areas, our results confirm the presence of extensive partisan segregation in the United States and show that offline partisan isolation, both considering physical encounters or residential sorting, is higher than online segregation and is primarily associated with educational attainment. Our findings emphasize the importance of physical space in understanding the relationship between social networks and political behavior, in contrast to the intense scrutiny focused on online social networks and elections.

Significance Statement In recent years, online social media have garnered growing attention for their influence on political polarization and, consequently, on election outcomes. As US society remains highly polarized, the relative contribution of online and offline social networks to this phenomenon is still debated. We compare social networks in physical and online spaces to understand the relationship between partisan exposure and vote choice at both the county and individual levels. We find that physical social networks better predict political outcomes, outweighing online ties and residential sorting, and we find partisan segregation to be higher in the physical space than in the online realm. Our results suggest a more fundamental role of real-world social mixing than online networks in reflecting political views.

## Introduction

Affective polarization has emerged as a critical concern for contemporary democracies, dividing populations along partisan and political lines and impacting various aspects of individuals’ lives (1–5). Moreover, recent years have witnessed considerable attention to the impact of online social media and news consumption—specifically misinformation and disinformation—on opinion dynamics, political polarization, and, consequently, on political elections (6–14). However, political polarization and partisan exposure cannot be solely explained by human behavior on social media platforms, as they are influenced by various dimensions of individuals’ daily lives, including physical and online environments, the role of social networks, and existing social divisions.

Homogeneous social networks can contribute to increased affective polarization and the maintenance of social and electoral cleavages through interactions with like-minded individuals (2, 15). In physical spaces, the debate has focused on the division of partisans at the residential level, where the grouping of like-minded people has increased at the geographical level in the United States (16–18). Specifically, individuals generally experience limited exposure to outgroup partisans, even within the same city or neighborhood (18). Furthermore, geographical sorting is significant both within and between geographical units (17), with larger differences observed within areas than between them (19). However, in terms of residential mobility, although Americans prefer politically compatible communities (20), they do not relocate for political reasons (21). Additionally, when considering individual mobility behavior, experienced partisan segregation in activity spaces varies depending on the physical space and geographic area (22), and online connections between geographic areas are driven by partisan homophily (23). Finally, individual survey data show that political polarization has increased in association with offline social network homophily (24). On the other hand, in online spaces and social media, affective polarization may be associated with self-selection and echo chambers (11, 25) or with the exposure to nonlocal interactions that may fuel political conflicts (14). However, ideological segregation in the United States is higher in face-to-face interactions than in offline and online news consumption (26), and partisan segregation in news consumption appears to be higher in traditional media than in online social networks (13, 26), with greater heterogeneity in partisan exposure online (27). Moreover, polarization has increased most significantly among social and demographic groups that are less likely to use social media (10), while deactivation experiments have shown no significant effects on voter turnout in US presidential elections or affective and ideological polarization (7).

Although previous studies have focused mainly on partisan exposure in terms of residential locations, experienced social exposure extends beyond the place of residence and involves routines, habits, and social interactions throughout people’s daily experiences (22, 28–32), both in physical and online spaces (33). In this context, social networks represent and shape many aspects of individual lives, with relevant impacts on access to novel information (34–36), economic actions and prosperity (37–39), and political participation (40–42). In particular, the literature highlights how social networks, including spouses, family, close friends and acquaintances, and the social environment foster political discussion and participation (43–47), and reinforce political beliefs (40, 48, 49). Moreover, some studies have shown that individuals’ vote choices are associated with the voting preferences of their political discussion networks (48, 50). Social exposure and its influence on political behavior is represented by both context and contact. Intergroup contact varies in depth, from casual to cooperative or selective encounters, and duration, from brief to sustained exposure (51). While most studies focus on cooperative and sustained interactions and draw on contact theory (52), research shows that proximity and casual exposure can also have significant effects on political behavior (53, 54). Understanding partisan social exposure and the dynamics of segregation in both physical and online dimensions is crucial to studying the transmission of political choices through social networks, following social influence and voting contagion (55, 56). However, a large-scale comparative analysis between online and physical partisan exposure in social networks and their relationship with political behavior is still lacking.

In this context, our study aims to understand the relationship between partisan exposure in online and physical spaces and voting outcomes. To this end, we integrate large-scale aggregated network data and individual surveys. Specifically, we first investigate whether partisan exposure differs across physical proximity and exposure to the same social context, online social connections, and residential sorting, both in metropolitan and nonmetropolitan areas. Second, we explore how partisan segregation at the county level relates to the demographic and socioeconomic characteristics of US counties. Third, we evaluate the dimension that best predicts voting patterns at both the county and individual levels. Our study is not intended to predict the results of future presidential elections. Instead, it focuses on contrasting existing online social networks with those in the physical world to describe electoral patterns.

To compare the online and physical exposure patterns, we leverage four datasets. First, we consider offline social connectedness using the Colocation Maps dataset, which provides the co-location probability between two randomly selected individuals residing in different US counties (57). Second, we analyze the Social Connectedness Index (SCI), which measures the relative probability of friendship on Facebook between two randomly selected individuals who reside in different counties (58). Third, we consider the probability of being exposed to Democratic and Republican voters based on the place of residence, looking at the neighbors of the individuals, using a publicly available dataset (59). We compute partisan exposure to Democrats and Republicans for each county in the contiguous United States and each dimension: offline, online, and residential. We then model the relationship with voting patterns at the county level and seek to determine which dimension best explains the observed variance of voting patterns. Fourth, we used the 2020–2022 Social Media Study data (60) provided by the American National Election Studies (ANES) to model the relationship between partisan exposure to Democrats and Republicans and the political vote in the 2020 presidential elections at the individual level. These datasets capture different types of contact across distinct contexts (see Table S1). Colocation Maps reflect physical co-presence, including both casual and selective, as well as brief and repeated encounters. These contacts are generally passive. The Social Connectedness Index captures digital social ties, typically selective, sustained, and active. Residential proximity represents the physical context in which all types of contact, casual or selective, brief or sustained, can occur. This form of contact is largely passive, shaped by where people live. Finally, survey data provide detailed measures of interpersonal relationships, reflecting selective, sustained, and primarily active forms of social contact.

Our findings reveal the strong and significant role of physical proximity and local exposure in predicting the voting outcomes of the US counties, as well as the role of offline ties in predicting individuals’ political votes in the 2020 presidential election when compared to online ties. Moreover, we find that partisan segregation is higher in offline social networks than in online ones, with physical partisan segregation primarily associated with the county population’s educational attainment and the urban–rural divide. Overall, our work contributes to a deeper understanding of how social exposure reflects political outcomes in the United States, specifically the voting patterns in presidential elections at both the county and individual levels, highlighting the effects of partisan segregation on voting polarization, with potential implications for affective polarization. Our results highlight the importance of gauging the relative role of different types of exposure, offline and online, when considering their effects on election results.

## Results

In this study, we compare offline and online partisan exposure and its relationship with vote choice at both the county and individual levels in the United States, and we examine partisan segregation. We first measure partisan exposure at the county level. Second, we analyze how partisan segregation relates to individuals’ demographic and socioeconomic characteristics within metropolitan areas. Finally, we estimate the relationship between partisan exposure and vote choice at both the county and individual levels.

### Defining partisan exposure across different dimensions

**Fig. 1.**
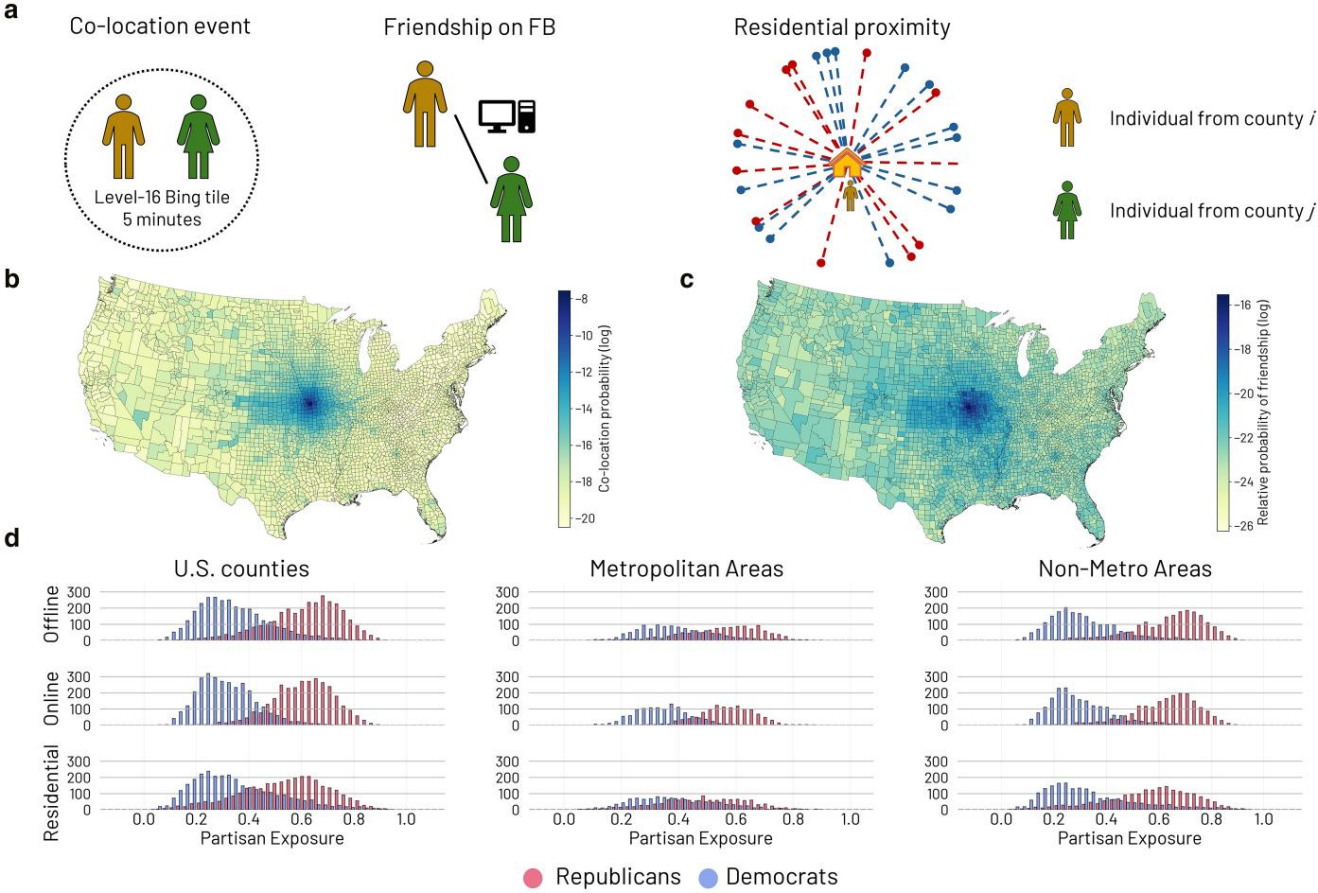
The three dimensions considered to estimate partisan exposure. a) A co-location event between two randomly selected individuals from counties *i* and *j* is defined as being co-located in the same place for at least 5 min, while the Social Connectedness Index accounts for the number of friendships on Facebook between individuals from *i* and *j*. Residential proximity considers the nearest 1,000 individuals that registered to vote. b and c) Co-location probabilities and relative probabilities of friendship on Facebook, respectively, between Jackson County, MO, and all the others. d) Distributions of partisan exposure by county, including metro and nonmetro areas. Note: distributions are not population-weighted.

We first compute the relative exposure to Republican and Democratic voters for each dimension. Physical partisan exposure is derived from the Colocation Maps, while online exposure is measured from the SCI. Specifically, the former provides the co-location probability (Fig. 1a) between two randomly selected individuals from US counties *i* and *j* (57). A co-location event (Fig. 1a) is registered when two Facebook users are at the same location for at least 5 min (57). In contrast, the SCI dataset provides the relative probability of friendship on Facebook between two users from *i* and *j* (Fig. 1a) (58). We refer to the Materials and methods section for further details.

Despite a high correlation between Colocation Maps and SCI raw probabilities (Pearson’s ρ=0.83), their corresponding networks show significant structural differences. Specifically, as shown in Fig. 1b and c, which compare the connection probabilities for Jackson County, MO, online connections are more homogeneously distributed across the country than offline co-location events. This is further explored in Fig. S1, which shows that US counties display a higher heterogeneity of social connections (network diversity D(i)) in the online space, with a median value of 0.39 compared to D(i)=0.15 offline. Moreover, US counties have higher online extroversion (defined as the ratio between external and internal connection probabilities) than offline extroversion, with a median value of 1.19 compared to 0.27.

Experienced physical and online partisan exposures, PE, for a county *i* are computed as the relative exposure to Republican and Democratic voters of a county *j* weighted by either the co-location or online connection probabilities between *i* and *j* see Materials and methods section for more details). Specifically, we define the share of Republican and Democratic voters as the average share of votes for Republican and Democratic candidates in three past presidential elections (i.e. 2012, 2016, and 2020), following the normal vote concept (61) to account for candidate- or election-specific influences on voter turnout and election results. Partisan exposure (PE) is a measure that ranges from 0 (low exposure to Democratic or Republican voters) to 1 (high exposure to either Democratic or Republican voters).

We then calculated the residential partisan exposure for each county from the dataset provided by R. Enos (18, 59), which provides the conditional probabilities of being exposed to Republican or Democratic voters for both Republicans and Democrats at the residential level, based on proximity to the nearest 1,000 individuals who have registered to vote. To the aim of our study, we compute the partisan exposure to Democratic and Republican voters for each US county as the probability that a random individual is exposed to Republicans or Democrats. Therefore, while measures of offline and online exposure are calculated from network ties between counties based on their political vote, residential exposure is derived from voter registration data. Further details can be found in the Materials and methods section.

To investigate the differences between partisan exposure across the three dimensions, we performed t-tests and Kolmogorov–Smirnov tests between the distributions of offline, online, and residential exposures (Table S2). Note that the distributions are not population-weighted. Considering all counties in the contiguous United States (Fig. 1d), we find significant differences (p<0.001) in partisan exposure to Republican voters when comparing residential exposure to online or offline exposure. However, no significant difference (p>0.05) is observed between the means of the distributions of online and offline exposures (Table S3). For Democratic voters, the differences in partisan exposure in the various dimensions are also significant (p<0.001). Finally, we categorize counties into metropolitan and nonmetro areas based on the Rural-Urban Continuum Codes (RUCC) and test the significance of the differences between partisan exposures (Fig. 1d; see Tables S4, S5, and S6 for complete results).

### Partisan segregation across demographic and socioeconomic factors

To investigate the levels of social segregation across political lines in the United States, we estimate partisan segregation for each dimension as the net difference between exposure to Republicans and exposure to Democrats in a given county. The metric ranges from −1 (indicating exclusive exposure to Democrats) to 1 (indicating exclusive exposure to Republicans) and accounts for third-party voters. Intermediate values represent different mixing levels, with 0 indicating an equal share of exposure between the two electorates (see Materials and methods section for further details). We then investigated the relationship between partisan segregation and the demographic and socioeconomic characteristics of the county, focusing on metropolitan areas. To this end, we predict partisan segregation for each network dimension using Gradient Boosting (GB) regressions, based on a set of demographic and socioeconomic factors: the shares of Hispanics and Latinos, African Americans, graduated individuals, unemployment, and urban population. Furthermore, we improve the explainability of the model by computing SHAP values (62), which provide importance scores that represent the impact of each variable on the predicted outcome. We refer to Materials and methods for further details on machine learning models.

**Fig. 2.**
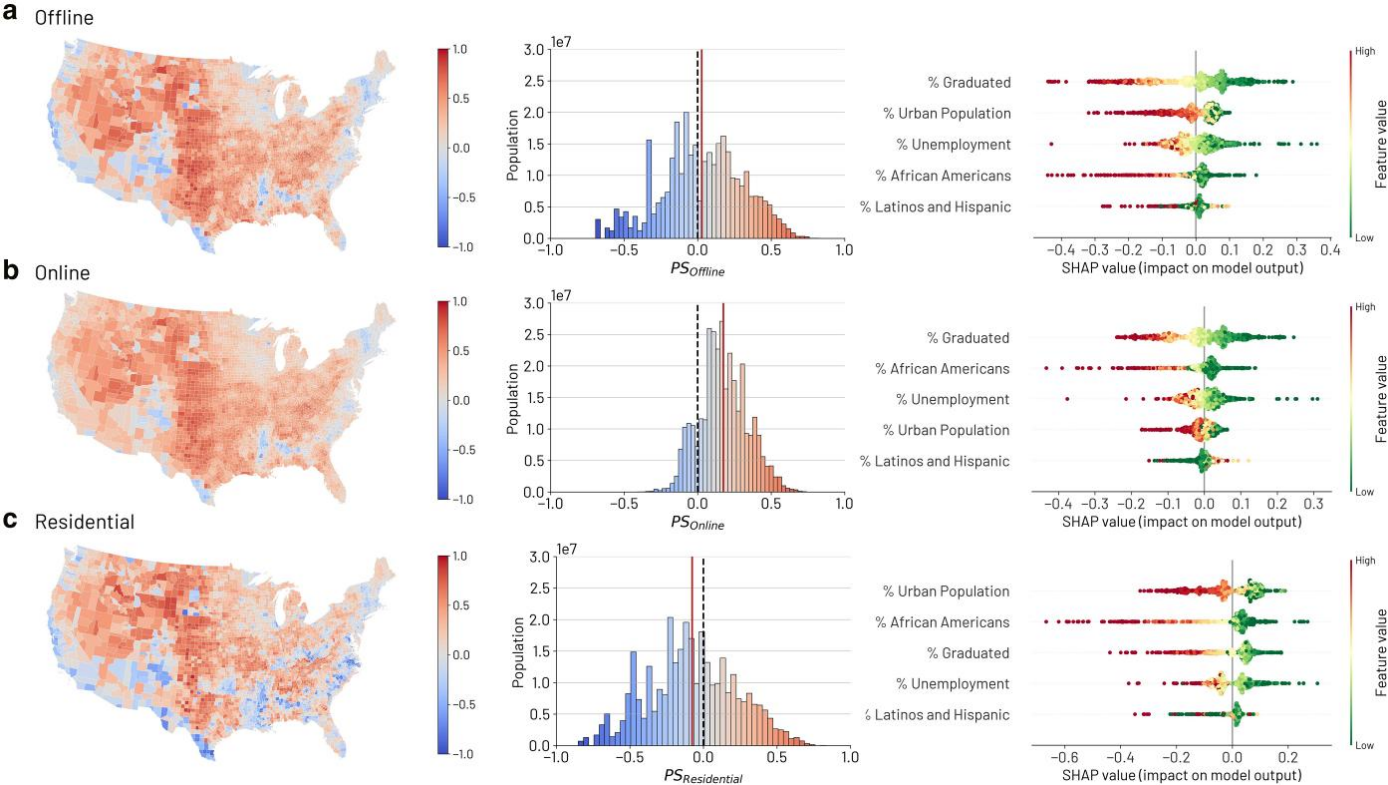
Partisan segregation across demographic and socioeconomic factors for each dimension. Maps and population-weighted distributions of the partisan segregation as captured by the Colocation Maps a), SCI b), and at the residential level c). The solid line represents the weighted average, while the dashed line indicates balanced social mixing (0). For each dimension, the SHAP values distributions, computed from the GB regressions, highlight how the demographic and socioeconomic characteristics of the counties impact partisan segregation. Predictors are ordered by their impact on the final prediction, with points colored red for high and green for low values.

Overall, partisan segregation in the physical world, both considering co-location maps and residential sorting, is higher than online segregation. As shown by the distributions of Fig. 2, in the offline and residential networks we observe counties characterized more frequently by PS>0.5 (highly Republican-segregated) or PS<−0.5 (highly Democrat-segregated). In particular, largely populated urban areas display values of PS<−0.5 in offline networks that are not met by online links.

In general, GB regressions effectively explain the variance of offline segregation ($R^{2}=0.63$), online (0.55), and residential (0.64). Moreover, Fig. 2 shows the distribution of SHAP values for each predictor related to offline, online, and residential partisan segregation in metropolitan areas. The predictors are ordered according to the impact on the prediction, with the most impactful variable at the top, and each point represents an observation. Finally, each point is colored according to its value, with red indicating high values and green indicating low values. The absolute impacts of the predictors are reported in the Table S7.

The percentage of graduated individuals is the best predictor for both partisan segregations computed from offline networks (average absolute impact equal to 0.096) and online (0.067) networks. While counties with higher shares of graduated individuals tend to be exposed to Democrats (with higher Democratic segregation), counties with less educated individuals tend to be co-located and connected online with individuals who vote for the Republican party (Fig. 2a and b). In contrast, at the residential level, the educational level follows similar patterns but is the third predictor (mean average impact equal to 0.067) in terms of variable importance (Fig. 2c).

Moreover, we find that the shares of urban population have a greater impact on partisan segregation in offline spaces, including both co-location (0.033) and residential (0.045) exposures, compared to online connections (0.016). Specifically, metropolitan areas with urban characteristics show higher levels of Democratic segregation in offline spaces (Fig. 2).

Furthermore, partisan segregation is closely related to the presence of ethnic communities. Specifically, the presence of a large percentage of African Americans is the second most relevant predictor of partisan segregation both online (0.037) and at the residential level (0.075) in metropolitan areas. Large African American communities are associated with predominant exposure to Democrats, while areas with a low African American presence tend to Republican segregation (Fig. 2). Finally, as shown in Fig. 2, the presence of Latinos and Hispanics is the least relevant predictor on all dimensions, namely offline (0.022), online (0.017), and at the residential level (0.029). Interestingly, a larger share of Latinos and Hispanics in US counties are associated with higher levels of Democratic and Republican offline and online segregation.

### Offline partisan exposure better predicts the vote choice of counties

We estimate the relationship between physical, online, and residential partisan exposure and vote choice through several statistical models to assess their relative contribution to voting dynamics. As with the calculation of partisan exposure, voting patterns of US counties are defined as the average share of Democratic and Republican voters in the last past presidential elections (i.e. 2012, 2016, and 2020). The shares are not complementary because we account for third-party and null votes. According to the normal vote concept (61), this method adjusts for candidate- or election-specific influences on voter turnout and election results, providing a more accurate assessment of voting patterns. In all the models, the shares of Democrats and Republicans in the US counties are our dependent or target variables.

We first model each dimension of partisan exposure (PE) and voting patterns for Democrats and Republicans separately with spatial autoregressive lag models (63), accounting for spatial autocorrelation across all counties in the contiguous United States. We design distinct models due to the high correlation between the dimensions, as shown in the Fig. S2. We use k-nearest neighbor to compute spatial weights, with different values of *k* (5, 7, and 10) for robustness. Similarly, we evaluate the relationship between partisan exposure and voting patterns in metropolitan and nonmetropolitan areas with ordinary least squares (OLS) regressions. Finally, we measure direct, indirect, and total effects of the independent variables for spatial models (see Table S11 and Fig. S3a), as well as marginal effects for metro and nonmetro areas (Fig. S3b and c). See Supplementary information for complete results (Tables S8 to S19).

**Fig. 3.**
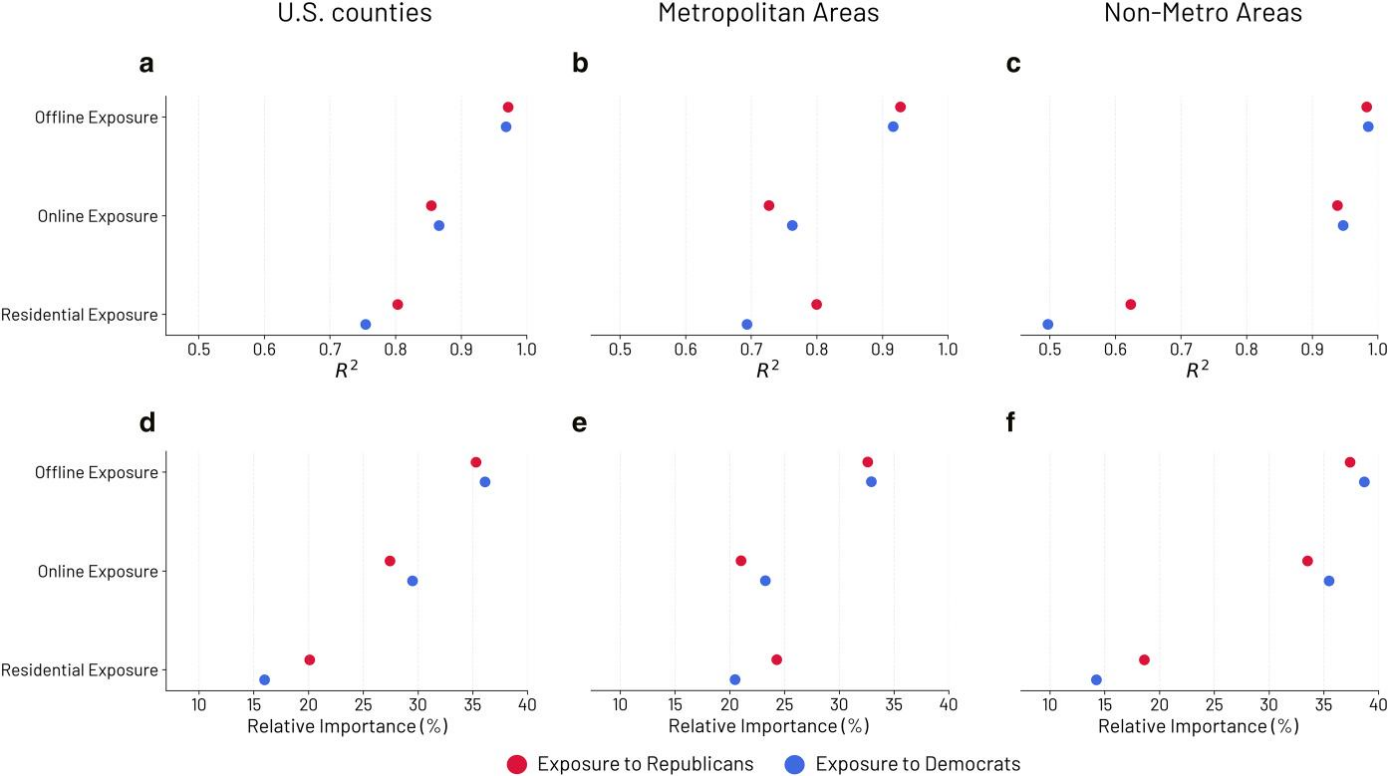
Relative contribution of the three dimensions of partisan exposure on voting patterns. Physical partisan exposure outweighs online and residential exposure considering all the counties in the contiguous United States in both spatial models a) with k=7 ($R^{2}$) and dominance analysis d) which considers demographic and socioeconomic controls. The result is consistent in both metropolitan b and e) and nonmetro areas c and f), employing both OLS models ($R^{2}$) and dominance analysis.

In Fig. 3, we show the results for both spatial and OLS models, for Democrats and Republicans, further disaggregated by metropolitan and nonmetropolitan counties. We compare the models’ ability to explain the variance in the dependent variable using $R^{2}$, with consistent results when measuring the model’s quality with the Akaike Information Criterion (AIC) and log-likelihood. Considering all US counties and accounting for spatial autocorrelation, physical partisan exposure to both Democrats and Republicans, as captured by Colocation Maps, is the dimension that best explains the variance in the share of Democratic ($R^{2}=0.97$) and Republican ($R^{2}=0.97$) votes, respectively, compared to online ($R^{2}=0.87$ and 0.85) and residential ($R^{2}=0.80$ and 0.75) exposures (Fig. 3a). Moreover, when comparing online and residential partisan exposures, the former has a higher explanatory power of the variance in voting patterns than the latter for Democrats and Republicans. Disentangling the analysis across the urban–rural axis according to the RUCC, we find that while in nonmetro areas offline and online partisan exposures have similar predictive power ($R^{2}=0.98$ and 0.94, respectively for Republicans and $R^{2}=0.99$ and 0.95 for Democrats), in metro areas the partisan exposure captured by Colocation Maps significantly outperforms both the other dimensions, with the $R^{2}$ of the model related to offline exposure equal to 0.93 (Republicans) and 0.92 (Democrats) compared to online (0.73 and 0.76, respectively) and residential (0.80 and 0.69) exposures (Fig. 3b and c). Therefore, in metropolitan areas, voting patterns are predominantly associated with partisan exposure to Democrats and Republicans in the physical space, when sharing the same social context.

To verify the robustness of our results, we test our findings with different modeling approaches. First, we perform a dominance analysis (64) to compare the three dimensions, taking into account possible demographic and socioeconomic confounders, including the shares of Hispanics and Latinos, African Americans, graduated individuals, unemployment, and urban population. These characteristics are selected based on the literature on the determinants of political outcomes (65–69) and by computing the variance inflation factors (VIF) to address multicollinearity issues. We refer to the Materials and methods section for further details about the variable selection. From this perspective, dominance analysis allows us to deal with highly correlated variables (Fig. S2) and determine the relative importance of each predictor. Figure 3d confirms the results obtained using the spatial lag models (Fig. 3a), with the higher relative importance of offline exposure in explaining the variance of US voting patterns. Specifically, offline exposure shows greater relative importance for both Republicans (35.29%) and Democrats (36.12%) compared to online (27.43 and 29.5%, respectively) and residential (20.11 and 15.97%) exposures. Moreover, the results of the OLS models (Fig. 3b and c) are confirmed by the dominance analysis in both metropolitan (Fig. 3e) and nonmetropolitan areas (Fig. 3f).

Second, we improve the generalizability of our findings by modeling the relationship between the three dimensions of PE and the voting patterns using Random Forest and Elastic Net models with k-fold cross-validation (70) (see Materials and methods section and Tables S20 and S21). Consistently with previous results, physical exposure, as captured by Colocation Maps, has the greatest impact on prediction compared to online and residential exposures. The findings hold when considering both the variable importance in the Random Forest models and the coefficients of the Elastic Net models (see the Section S7). In general, the models achieve high performance in terms of generalizability, with $R^{2}$ between 0.97 and 0.98 in the test sets for all models.

To compare our results with official statistics, we calculated partisan exposure using the 2016–2020 5-Year ACS Commuting Flows and modeled the relationship between partisan exposure and the voting patterns of the US counties. Partisan exposure, as captured by commuting flows, outperforms online and residential proximity in predicting voting patterns but shows lower performance compared to physical exposure, as confirmed by both spatial models and dominance analysis (Tables S22 to S25 and Fig. S4).

Finally, as a sensitivity analysis, we excluded the self-loops from the co-location and online connections networks. A self-loop represents the co-location or friendship probability between individuals in county *i* and residents of the same county, leading to an edge between *i* and itself. In the physical network, local exposure represents a substantial and fundamental aspect of an individual’s exposure, with a median value of counties’ extroversion equal to 0.27. In contrast, while local exposure still plays a significant role in the online network, the network structure is more heterogeneous and characterized by higher external exposure (median value of extroversion equal to 1.19). Detailed descriptions can be found in the Supplementary information (Fig. S1). Figure S5 shows that when local exposure is excluded from the calculation of PE, online ties outweigh physical proximity in predicting US political outcomes at the county level. Specifically, considering all the US counties, online exposure explains 65 and 67% ($R^{2}$) of the variance of voting patterns for Republicans and Democrats, respectively, compared to 61 and 60% explained by the physical proximity (Fig. S5a). However, predictive performance is lower when local exposure is not considered, particularly in the offline network, characterized by low heterogeneity and extroversion. Hence, local exposure appears to be a fundamental dimension in predicting voting outcomes.

### Offline partisan exposure better predicts individuals’ vote choice

We combine large-scale analysis with individual survey data to better understand the relationship between partisan exposure in physical and digital spaces and vote choice. To this end, we leverage the 2020–2022 Social Media Study dataset (60) provided by the American National Election Studies (ANES), which consists of an online survey panel conducted in three waves (pre- and post-election 2020, and the 2022 midterm elections). As explained in the Materials and methods section, our focus is on the first two waves, including all respondents who declared their vote choice in the 2020 presidential election for either the Democratic or Republican party, had a valid vote, and responded to the following questions: “How many of your friends and family are Democrats?,” “How many of your friends and family are Republicans?,” “How many of your Facebook friends are Democrats?,” and “How many of your Facebook friends are Republicans?.” The final sample consists of 2,420 respondents. Descriptive information about the variables can be found in the Supplementary information (Table S26).

As described in the Materials and methods section, we performed four logit models to distinguish between exposure to Democrats and Republicans and to account for each wave (pre- and post-election). The dependent variable is vote choice, encoded as a binary variable with 0 for the Democratic party and 1 for the Republican party. We control for the age, ethnicity, educational attainment and place of residence of the respondents (metro or nonmetro area), and compute and apply post-stratification weights based on gender, age, education and ethnicity using the 5-year estimates 2020–2024 of the American Community Survey (ACS). Detailed results can be found in the Supplementary information (S27).

**Fig. 4.**
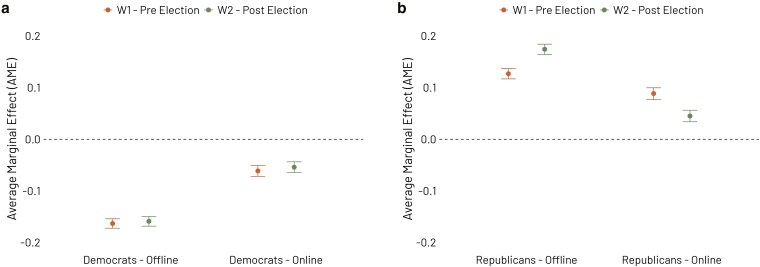
Average marginal effects of partisan exposure (online and offline) on vote choice. Offline partisan exposure has a stronger average marginal effect on vote choice than online partisan exposure, both in terms of exposure to Democrats (a) and Republicans (b). Logit models control for respondents’ age, ethnicity, educational attainment, and place of residence (metro or nonmetro area). The dependent variable is binary, with 0 for voting Democrat and 1 for voting Republican.

Figure 4 shows the average marginal effects (AME) of offline and online partisan exposure on vote choice across the two waves. As shown, offline exposure to Democrats has a stronger effect on voting for the Democratic party than online exposure (4a), while offline exposure to Republicans more significantly impacts voting for the Republican party (4b). Specifically, in the first wave, the average marginal effect of the exposure to Democrats is −0.163 offline compared to −0.061 online, while in the second wave it is −0.159 compared to −0.054. Similarly, related to the exposure to Republicans, the average marginal effect is 0.127 offline compared to 0.089 online in the first wave, and 0.174 compared to 0.045 in the second wave.

## Discussion

In this study, we compared partisan exposure across physical and online spaces, evaluated the association between partisan segregation in online and physical spaces and the underlying counties’ demographic and socioeconomic characteristics, and investigated the relationship between partisan exposure and vote choice at both the individual and county levels. Our findings highlight the dominant role of experienced physical proximity and local exposure in predicting vote choice in the United States at both the individual and county levels, outperforming the influence of online ties and residential proximity. The result was consistent across both metropolitan and nonmetro areas. Moreover, we found that partisan segregation is higher in offline social networks than online ones, and such a difference is primarily driven by individuals’ educational attainment. The urban–rural divide significantly shapes offline and residential partisan segregation, with Republican voter segregation more prevalent in metro areas with large rural populations. Thus, physical partisan exposure, which is the best predictor of voting outcomes, is also strongly associated with educational attainment and urbanization levels. From this perspective, our findings contribute to a better understanding of the relationship between social networks, physical and online spaces, and political behavior, highlighting the effects of partisan segregation on voting polarization, with potential implications for affective polarization (1).

Despite significant attention to the impact of new media technologies, particularly online social media, on affective polarization and political elections (6–14), either through mechanisms of echo chambers and selective exposure (11, 25) or through interactions outside of one’s local bubbles (14), our findings highlight the greater relevance of physical space and offline ties in predicting political behavior. This is demonstrated through both large-scale (i.e. county-level) and individual survey analyses, enhancing our understanding of the relationship between partisan exposure and political vote. From this perspective, offline social networks, local exposure, and physical proximity, characterized by low heterogeneity of social exposure and low extroversion, better reflect vote choice at the individual and county levels compared to online ties. Similar to prior research (53, 54), our results suggest that even brief, passive, and casual forms of exposure, which can be captured by co-location events, can influence or be associated with political behavior. Therefore, beyond the form of exposure, context matters, with offline contexts outweighing online exposure. In line with the literature that examines the association between social networks and vote choice (50, 71), these findings confirm the link between homogeneous social networks and political vote, particularly in physical space, where Americans experience greater partisan segregation. This underscores the persistent influence of offline social ties compared to online ones, despite increasing attention to social media platforms and their potential impact on vote choice and affective polarization.

In general, partisan exposure and segregation vary between physical and online spaces, as well as between urban and rural areas. In line with the literature, our findings highlight that up to 10% of the American population experiences a low exposure to outgroup partisans, not only in the residential sphere (18), but also in physical social encounters. Our analysis shows that experienced segregation is even greater than online partisan segregation, consistent with previous studies (13, 26, 27). Both forms of experienced segregation are associated with the educational attainment of individuals, a known driver of socioeconomic segregation (72). Furthermore, the tendency of Republican voter segregation in areas with large rural populations reveals differences between urban and rural areas related not only in terms of political party support (73) but also in partisan exposure (18). In contrast, consistent with the literature, our findings show greater Democratic segregation in high-density urban areas (18).

Our study comes with some limitations. First, we compared online and physical networks using aggregated observational data from social media platforms. Despite the two-step reweighting applied to Colocation Maps (57), the Facebook data might underrepresent certain categories of people (e.g. young and old individuals) and the population of rural areas (74). Moreover, these aggregated networks are considered at a rather coarse geographical level, the US county, whose population sizes vary widely, ranging from 83 individuals (Loving County, TX) to over 10 million inhabitants. Furthermore, we use the SCI as a proxy of online connections, but people interact through multiple social media platforms and have access to novel information from different sources. However, although some studies have attempted to spatially map online interactions (33, 75, 76), achieving comprehensive coverage throughout the United States remains challenging. Moreover, we acknowledge that co-location does not imply a face-to-face interaction between individuals. However, co-presence temporal networks have been found to have structural and statistical features comparable to face-to-face interaction temporal networks (77). Additionally, individuals’ partisan exposure, as measured by the ANES Social Media Study both before and after the 2020 US presidential election, reflects self-perceived partisan exposure and may be subject to self-report biases, such as social desirability or recall inaccuracy. Finally, as mentioned, these datasets capture different types of contact, varying in duration, selectivity, and the extent to which they are active or passive. This is also reflected in the mismatch between county- and individual-level analyses, with the latter capturing more active forms of exposure, typically driven by friendship or kinship ties. Future work should aim at measuring the partisan balance of individual’s social networks, online and offline, based on one comprehensive data source.

In conclusion, our study underscores the centrality of physical space in understanding human and political behavior, with potential implications for affective and voting polarization driven by partisan segregation. Despite challenges related to data limitations and privacy concerns, future studies on political polarization, social networks, and partisan segregation, and their impact on political outcomes, should not overlook the importance of real-world interactions in physical space.

## Materials and methods

### Data sources

#### Online network

We use the SCI (58) dataset provided by Meta through its Data for Good program as a proxy of online connections. The SCI measures the social connectedness between administrative areas by computing the probability at which two random individuals, living respectively in administrative areas *i* and *j*, are friends on Facebook, as of April 2016 (58). The SCI is available at the county and zip code levels. We analyze online connections at the county level to enable comparison with the Colocation Maps. Specifically, to be able to compare the online probabilities with the offline ones, we use the original version of the dataset adopted in Bailey et al. (58), which includes the relative probabilities of friendship between counties computed as follows:


(1) $$rel\_prob\_friend=C\frac{\;f_{ij}}{pop_{i}\;*\;pop_{j}},$$

where $f_{ij}$ is the number of Facebook friendships between administrative regions *i* and *j* and $pop_{i}$ and $pop_{j}$ are the population size of *i* and *j*. *C* is a scaling factor, equal to $10^{12}$, which we drop to obtain the raw probabilities.

#### Offline network

We use the Colocation Maps (57) provided by Meta through its Data for Good program to measure offline exposure. Similarly to the SCI, the dataset provides the weekly co-location probability between two randomly selected individuals from two administrative areas *i* and *j*. A co-location event (Fig. 1a) is registered when two individuals who enabled location-based services on the Facebook smartphone application are located in the same place (i.e. a level-16 Bing-tile, approximately 600 by 600 meters at the Equator) at the same time for at least 5 minutes. The co-location probability between geographic regions *i* and *j* is then determined by the number of co-location events between individuals from *i* and *j* weighted by their population sizes. As described by Iyer et al. (57), the authors applied a two-step reweighting to enhance representativeness. Specifically, the first step aligns the Facebook user data with the general population, and the second step reweights the Colocation Maps population to the Facebook user base. The dataset has been designed and used mainly for epidemiological purposes (78, 79). For the aim of our study, we collected weekly co-location probabilities between US counties from August 2022 to January 2024, for a total of 76 weeks/observations. We then use the average co-location value over the full period to mitigate seasonality effects and data sparsity and reduce computational errors in data measurement, ensuring a more realistic measure of physical exposure between counties.

#### Residential partisan exposure

We compare online and offline networks with a measure of residential proximity, based on the residential locations of individuals. To this purpose, we use the dataset provided by R. Enos (59) supporting the recent study by Brown et al. (18), which includes the conditional probability of exposure to Democratic and Republican voters, conditioned on an individual’s political affiliation (either Democrat or Republican). The probabilities are computed based on the nearest 1,000 individuals, but the results remain consistent even considering a larger sample size of 50,000 individuals. The dataset is derived from voter registration records, which require individuals in the majority of US states to declare their party affiliation and it provides information at different geographical levels. To have a measure of partisan exposure at the residential level, we compute the probabilities of exposure to Democrats and Republicans for a random individual for each US county. Specifically, from the available raw data, we compute the partisan exposure to Republicans for a county *i* as follows:


(2) $$P(R)=\frac{P(R|D)P(D)}{P(D|R)},$$

where P(R|D) is the conditional probability of exposure to Republicans being a Democrat and P(D|R) is the conditional probability of exposure to Democrats being a Republican. Similarly, we compute the partisan exposure to Democrats.

#### ANES 2020–2022 social media study

We analyze individual-level exposure to Democrats and Republicans and their relationship with political voting in the 2020 presidential election using the 2020–2022 social media study provided by the American National Election Studies (ANES) (60). The study is based on an online survey panel of 3 waves (i.e. before and after the 2020 elections, and the 2022 midterm elections). We focus on the first two waves, which include 5,750 and 5,277 respondents, respectively. From this sample, we consider only those respondents who declared their political preference in the second wave (w2presvtwho), have valid voting records (vote20_match), and answered all the questions of interest. The final sample consists of 2420 respondents. Specifically, we focus on the following questions: “How many of your friends and family are Democrats?,” “How many of your friends and family are Republicans?,” “How many of your Facebook friends are Democrats?” and “How many of your Facebook friends are Republicans?” Each of these questions is measured on a scale from 1 to 5, where 1 indicates “None or almost none,” and 5 indicates “All or nearly all.” We then compute new post-stratification weights based on gender, age, education, and ethnicity using the 2020–2024 5-year estimates of the American Community Survey (ACS).

#### Voting patterns in US counties

We define the voting patterns of counties in the contiguous United States following the normal vote concept (61) to account for candidate- and election-specific influences on voting outcomes. Specifically, the share of Republican and Democratic voters for each county is computed as the average of three presidential elections (i.e. 2012, 2016, and 2020). We obtained data on presidential election results from the work by Algara and Amlani (80).

#### Demographic and socioeconomic indicators

To evaluate the relationship between partisan segregation and county characteristics, we obtain data from 5-year estimates of the American Community Survey (ACS) for 2017–2021 provided by the US Census Bureau (81). Specifically, we used data related to the share of Hispanics and Latinos, African Americans, unemployed individuals, and urban population. In addition, we use information about the share of graduated individuals for each county provided by Chetty et al. (82) through the Opportunity Insights repository (83). We select this information from a broader set of counties” characteristics by computing Variance Inflation Factors (VIFs) to address multicollinearity issues and drawing on the literature on the determinants of county outcomes in US presidential elections. Specifically, ethnicity is one of the strongest predictors of political outcomes, and African Americans tend to vote for the Democratic party (66, 68). Furthermore, educational attainment, which is highly correlated with income (addressed through VIF), is associated with Democratic voting tendencies (65, 66). Finally, unemployment is one of the economic indicators that affect political outcomes (67), as well as the characteristics and divide of urban–rural regions (69, 73).

#### Metropolitan and nonmetropolitan areas

We define metropolitan and nonmetropolitan areas based on the Rural-Urban Continuum Codes (RUCC) 2013 (84). Specifically, metropolitan areas include the codes 1 to 3 (RUCC 1–3), while nonmetropolitan areas include codes 4 to 9 (RUCC 4–9).

### Methods

#### Partisan exposure

We define the partisan exposure of a county *i* as the relative exposure to people with a certain voting behavior (i.e. Republican or Democratic voters), weighted by either the co-location probability between counties *i* and *j* (i.e. Colocation Maps) or the relative probability of friendship on Facebook between *i* and *j* (i.e. the SCI). Formally, we define the partisan exposure as:


(3) $$PE_{\;p}(i)=\sum_{\;j}^{N}v_{\;p}(\;j)\frac{\;p_{ij}}{\sum_{k}^{N},p_{ik}},$$

where ${\mathrm{PE}}_{\;p}(i)$ is the partisan exposure to either Republicans or Democrats for a county *i*, *N* is the number of US counties, $v_{\;p}(j)$ is the percentage of Republican or Democratic voters in the county *j*, and $p_{ij}$ is either the co-location probability between counties *i* and *j*, or the relative probability of friendship on Facebook between *i* and *j*. Partisan exposure ranges from 0 (low exposure to either Democrats or Republicans) to 1 (high exposure to either Democrats or Republicans). The sum of partisan exposures to Republicans and Democrats is <1 because we account for null and third-party votes.

#### Residential and experienced partisan segregation

Residential and experienced partisan segregation are computed from the residential and online or offline partisan exposures, respectively. Specifically, partisan segregation is computed as the difference between exposure to Republicans and Democrats. The index ranges from −1 (indicating exclusive exposure to Democrats) to 1 (indicating exclusive exposure to Republicans) and accounts for third-party voters (since the sum of ${\mathrm{PE}}_{\mathrm{rep}}(i)$ and ${\mathrm{PE}}_{\mathrm{dem}}(i)$ may be <1). Intermediate values indicate varying levels of partisan mixing. The index is computed as follows:


(4) $$\mathrm{PS}(i)={\mathrm{PE}}_{\mathrm{rep}}(i)-{\mathrm{PE}}_{\mathrm{dem}}(i),$$

where PS(i) is the index of partisan segregation for a county *i*, ${\mathrm{PE}}_{\mathrm{rep}}(i)$ is the partisan exposure to Republicans and ${\mathrm{PE}}_{\mathrm{dem}}(i)$ is the partisan exposure to Democrats.

#### Spatial autoregressive lag models and OLS

To explore which dimension of partisan exposure better predicts voting patterns in US counties, we separately model physical, online, and residential exposures using spatial regressions, both for Republicans and Democrats. Due to the high correlation between these dimensions (see Supplementary information S2A), combining them in the same model could impact interpretation and potentially lead to misleading conclusions. Specifically, we use spatial lag models (63), which are autoregressive models that account for spatial autocorrelation among observations. Specifically, the models are defined as:


(5) $$v_{\;p}(i)=\alpha +\rho Wy+\beta {\mathrm{PE}}_{\;p,d}(i)+\epsilon ,$$

where *v* is the share of either Republican or Democratic votes, *p* is either Democrats or Republicans, ρWy is the autoregressive coefficient, and PE is the partisan exposure to either Democrats or Republicans, *p*. The model is fitted for each dimension *d*, that is, offline, online and residential. To enhance the robustness of the method, we compute the spatial weights of k-nearest neighbors *W* using different values of *k*, specifically 5, 7, and 10. This approach is preferred because contiguity-based methods would exclude noncontiguous counties from the analysis. When comparing metropolitan and nonmetropolitan areas based on the Rural-Urban Continuum Codes (RUCC), we use ordinary least squares (OLS) regressions. In both types of models, we determine the dimension that better predicts voting patterns based on its ability to explain the variance of the dependent variable (i.e. $R^{2}$).

#### Dominance analysis

While we treat the three dimensions separately in the regressions, due to their high collinearity, we use the dominance analysis (64) to consider them together and evaluate their relative importance. This method allows us to evaluate the impact of predictors by measuring the contribution of each predictor through designing a series of linear regressions with all possible combinations of predictors to systematically assess and compare the individual and combined predictive power of each variable. Specifically, we perform a dominance analysis for both the exposure to Republicans and Democrats, including physical, online, and residential exposure in the models along with the demographic and socioeconomic characteristics previously described. The method provides the relative importance of each predictor as a percentage (Fig. 3). The full model is defined as:


(6) $$\begin{array}{rl} v_{\;p}(i)\;= & \;\alpha +\beta_{1}{\mathrm{PE}}_{\;p,\mathrm{offline}}(i)\\ & +\beta_{2}{\mathrm{PE}}_{\;p,\mathrm{online}}(i)\\ & +\beta_{3}{\mathrm{PE}}_{\;p,residential}(i)\\ & +\beta_{n}\mathrm{controls}(i)+\epsilon , \end{array}$$

where we also account for the demographic and socioeconomic characteristics of the counties.

##### Logit regressions on survey data

To understand the relationship between partisan exposure and vote choice, we model four logit regressions, differentiating between exposure to Democrats and Republicans for each wave (pre- and post-election). This approach accounts for the complementary nature and negative correlation between questions like “How many of your friends and family are Democrats?” and “How many of your friends and family are Republicans?” The logistic regression models are specified as follows:


(7) $$v(i)=\alpha +\beta_{1}{\mathrm{PE}}_{\mathrm{offline}}(i)+\beta_{2}{\mathrm{PE}}_{\mathrm{online}}(i)+\beta_{n}\mathrm{controls}(i)+\epsilon ,$$

where v(i) is the respondents’ vote at the 2020 presidential elections, ${\mathrm{PE}}_{\mathrm{offline}}$ represents offline partisan exposure based on friends and family voting behavior, and ${\mathrm{PE}}_{\mathrm{online}}$ represents online partisan exposure based on Facebook friends’ vote choice. We include controls for respondents’ age, ethnicity, educational attainment, and place of residence (metro or nonmetro area), and apply post-stratification weights based on gender, age, education, and ethnicity using the 2020–2024 5-year estimates of the American Community Survey (ACS). Ethnicity and educational attainment are treated as dummy variables, differentiating between the White, Black, and Hispanic categories and between individuals with and without a degree.

##### Predictive modeling and model explainability

To enhance the robustness and generalizability of the result related to the relationship between partisan exposure and voting patterns, we model each dimension using random forest and Elastic Net (70) models with k-fold cross-validation (k=5). This allows us to evaluate variable importance using tree models and beta coefficients of the model using a regularization technique.

Second, we predict partisan segregation in metropolitan areas (RUCC 1–3) for each dimension using demographic and socioeconomic characteristics of US counties. To this aim, we model Gradient Boosting regressions and compute SHAP (62) values to interpret the predictions. Specifically, drawing on game theory, the SHAP framework computes importance scores for each predictor, representing the impact of each variable on the final prediction. Before training the model, we split the dataset into training and test sets using a 70/30 ratio.

## Supplementary Material

pgaf308_Supplementary_Data

## Data Availability

Colocation Maps are available through the Meta Data for Good program by signing a data sharing agreement (https://dataforgood.facebook.com/). The Social Connectedness Index (SCI) is publicly available through the Humanitarian Data Exchange portal (https://data.humdata.org/dataset/social-connectedness-index). Residential partisan exposure is available through Harvard Dataverse (https://doi.org/10.7910/DVN/A40X5L). The ANES 2020-2022 Social Media Survey dataset is available at https://electionstudies.org/data-center/2020-2022-social-media-study/. The code for reproducing the study findings is available at https://github.com/tonmarco.

## Preprints

A preprint of this article is published at https://arxiv.org/abs/2407.12146.
