# Supplementary material for: Physical partisan proximity outweighs online ties in predicting US voting outcomes
Source: PNAS Nexus. 2025 Oct 21;4(10):pgaf308. doi: 10.1093/pnasnexus/pgaf308 (PMC12538564; doi:10.1093/pnasnexus/pgaf308)
Supplement: pgaf308_Supplementary_Data [file pgaf308_supplementary_data.pdf]

Supporting Information for

Physical partisan proximity outweighs online ties in predicting US voting outcomes

Marco Tonin, Bruno Lepri, Michele Tizzoni

This PDF file includes:

Figs. S1 to S5
Tables S1 to S27
SI References

Contents

1 Data sources and type of contacts or encounters 3

2 Diversity and extroversion in online and offline networks 4

3 Partisan exposure across dimensions 5
A Correlation between partisan exposure across dimensions 5
B Differences in partisan exposure across dimensions 6

4 Partisan segregation results 9

5 Regression results 10
A Spatial autoregressive lag models 10
B Direct, indirect, and total effects in spatial lag models 13
C OLS models for metro and non-metro areas 14
D Marginal effects of spatial and OLS models 16

6 Dominance analysis results 17
A Dominance analysis results for metro and non-metro areas 18

7 Random Forest and Elastic Net models 20

8 Comparison between Colocation Maps and ACS Commuting Flows 21

9 Exclude local exposure (self-loops) in the networks 26

10 Descriptive and regression analyses of survey data 27

1. Data sources and type of contacts or encounters

We compare partisan exposure across physical and digital spaces using datasets that capture different types of contact or encounters. Table S1 details the data sources, units of analysis, and what each represents. As discussed in the manuscript, although most of the literature has focused on cooperative and sustained contact, drawing on contact theory (1), even brief and casual encounters can influence political behavior (2–4).

Table S1. Data sources and types of contacts and encounters

Data Source	Unit of Analysis	Type of Encounter
Colocation Maps	Probability	Co-location for at least 5 minutes. It may be brief or sustained, casual or cooperative/selective. Generally passive.
Social Connectedness Index	Probability	Digital social ties on Facebook, typically selective, sustained, and active.
Residential exposure	Probability	Exposure to neighbors. All types of contact, casual or selective, brief or sustained, can occur. Largely passive.
ANES survey	Likert scale	Offline and online interpersonal relationships, reflecting selective, sustained and primarily active forms of social contact.

2. Diversity and extroversion in online and offline networks

The heterogeneity of social connectedness is defined as a function of Shannon's entropy. The measure provides the degree of diversity of counties' social connections, taking values from 0 (low diversity) to 1 (high diversity).

$$D(i) = -\frac{\sum_{j=1}^{k} p_{ij} \log(p_{ij})}{\log k}, \quad [1]$$

where k is the number of counties, and $p_{ij} = \frac{V_{ij}}{\sum_{j=1}^{k} V_{ij}}$ where V_{ij} is either the co-location probability between i and j, or the relative probability of friendship on Facebook between i and j.

The extent of external exposure is defined with a measure of extroversion by computing the ratio between external and internal probabilities for each county, both for the co-location and friendship networks. The measure is scaled between 0 (low extroversion) and 1 (high extroversion).

$$E(i) = \frac{p_{int}}{p_{ext}}, \quad [2]$$

where p_{int} is either the co-location or friendship probability between a county i and itself, and p_{ext} is the sum of either the co-location or friendship probabilities of the county i with all the other counties.

As shown in Fig. S1, the links in the online network are more heterogeneous compared to the offline network (See Fig. S1a). Additionally, network diversity is strongly correlated with the degree of extroversion in social connections for both online and offline networks (See Fig. S1b and S1c).

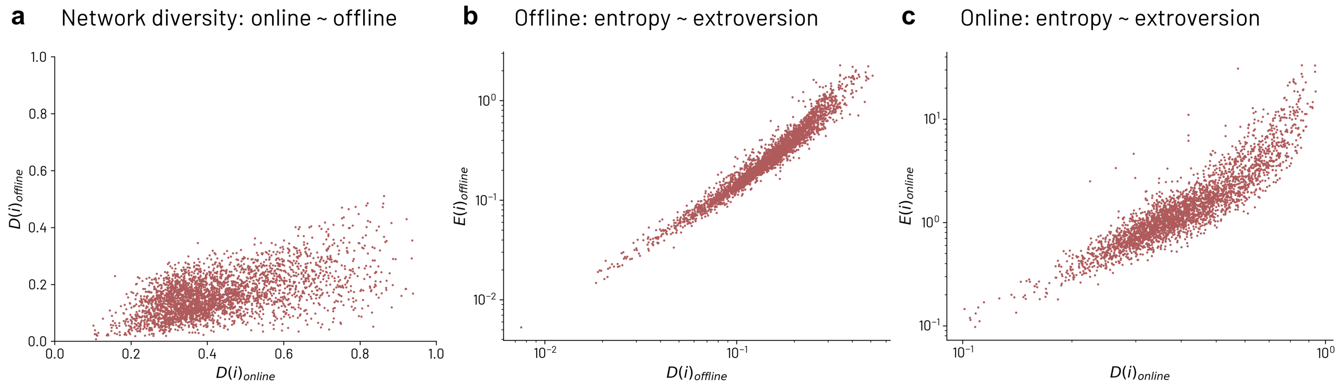

Fig. S1. Heterogeneity and extroversion in the online and offline networks. (**a**) Relationship between online and offline network diversity. (**b**) Relationship between offline network diversity and extroversion. (**c**) Relationship between online network diversity and extroversion.

3. Partisan exposure across dimensions

A. Correlation between partisan exposure across dimensions. As mentioned in the manuscript, partisan exposure across physical and online spaces are highly correlated. Specifically, as shown in Fig. S2, the correlation between online and offline partisan exposure is equal to 0.96 for Republicans and 0.97 for Democrats. The correlation between offline and residential is 0.81 and 0.74 respectively. Finally, the correlation between online and residential partisan exposure is 0.77 and 0.72 respectively.

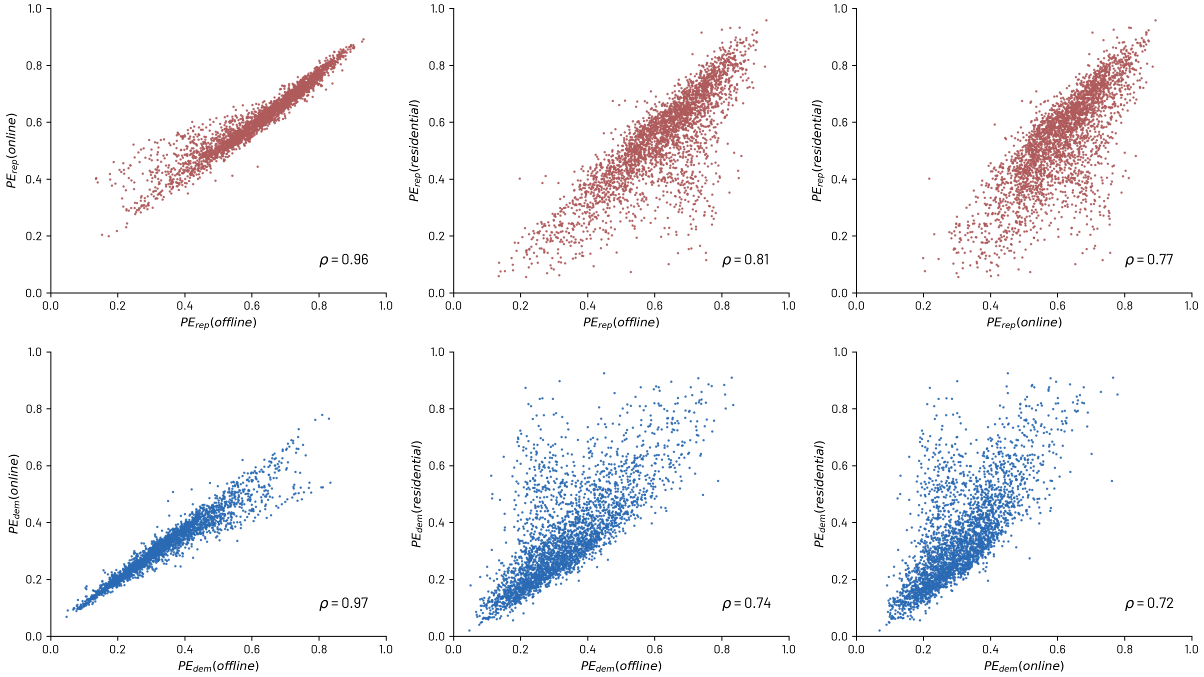

Fig. S2. Correlation between dimensions.

B. Differences in partisan exposure across dimensions. We assess the differences in partisan exposure across physical and online spaces, as well as between metropolitan and non-metropolitan areas. To this aim, we perform t-tests and Kolmogorov-Smirnov tests to compare the different dimensions and Welch's t-tests and Kolmogorov-Smirnov tests to evaluate the differences between metropolitan and non-metropolitan areas.

Table S2. Descriptive statistics of partisan exposure

Variable	Mean	SD	Min	Max
PE_{rep}(offline)	0.620	0.138	0.134	0.933
PE_{rep}(online)	0.613	0.112	0.200	0.892
PE_{rep}(residential)	0.541	0.166	0.057	0.958
PE_{dem}(offline)	0.334	0.133	0.047	0.834
PE_{dem}(online)	0.309	0.107	0.069	0.778
PE_{dem}(residential)	0.349	0.168	0.021	0.925

Exposure to	Dimension 1	Dimension 2	T-statistic	Significance	KS statistic	Significance
DEM	Offline	Online	7.950	***	0.099	***
DEM	Offline	Residential	-3.968	***	0.072	***
DEM	Online	Residential	-11.12	***	0.154	***
REP	Offline	Online	1.931	ns	0.093	***
REP	Offline	Residential	20.31	***	0.212	***
REP	Online	Residential	20.22	***	0.230	***

Note: $^{*}\,p < .05$; $^{**}\,p < .01$; $^{***}\,p < .001$

Table S3. Comparison of the dimensions for all the counties in the contiguous United States using t-test for the statistical significance.

Exposure to	Dimension 1	Dimension 2	T-statistic	Significance	KS statistic	Significance
DEM - Metro	Offline	Online	9.382	***	0.171	***
DEM - Metro	Offline	Residential	-0.991	ns	0.067	*
DEM - Metro	Online	Residential	-9.383	***	0.218	***
REP - Metro	Offline	Online	-2.205	*	0.122	***
REP - Metro	Offline	Residential	12.87	***	0.234	***
REP - Metro	Online	Residential	16.69	***	0.324	***

Note: $^{*}\,p < .05$; $^{**}\,p < .01$; $^{***}\,p < .001$

Table S4. Comparison of the dimensions in metropolitan areas using t-test for the statistical significance.

Exposure to	Dimension 1	Dimension 2	T-statistic	Significance	KS statistic	Significance
DEM - Non-Metro	Offline	Online	3.438	***	0.060	**
DEM - Non-Metro	Offline	Residential	-4.287	***	0.844	***
DEM - Non-Metro	Online	Residential	-7.351	***	0.119	
REP - Non-Metro	Offline	Online	4.011	***	0.102	***
REP - Non-Metro	Offline	Residential	16.69	***	0.237	***
REP - Non-Metro	Online	Residential	13.96	***	0.186	***

Note: $^{*}\,p < .05$; $^{**}\,p < .01$; $^{***}\,p < .001$

Table S5. Comparison of the dimensions in non-metropolitan areas using t-statistic for the statistical significance.

Exposure to	Dimension 1	Dimension 2	T-statistic	Significance	KS statistic	Significance
DEM	Offline - Metro	Offline - Non-Metro	15.92	***	0.279	***
DEM	Online - Metro	Online - Non-Metro	12.47	***	0.256	***
DEM	Residential - Metro	Residential - Non-Metro	10.20	***	0.226	***
REP	Offline - Metro	Offline - Non-Metro	-17.72	***	0.294	***
REP	Online - Metro	Online - Non-Metro	-16.08	***	0.301	***
REP	Residential - Metro	Residential - Non-Metro	-14.06	***	0.239	***

Note: $^{*} p < .05$; $^{**} p < .01$; $^{***} p < .001$

Table S6. Comparison between exposure in metropolitan and non-metropolitan areas using Welch's t-test for the statistical significance.

4. Partisan segregation results

As explained in the manuscript, we enhance model explainability related to the prediction of physical, online, and residential partisan segregation by computing the SHAP values. The absolute impact of the predictors on the final predictions are the following.

Variable	Offline	Online	Residential
% graduated	0.095842	0.067271	0.067291
% urban population	0.052936	0.022199	0.080493
% unemployed	0.048427	0.034916	0.056921
% African Americans	0.037656	0.037174	0.074869
% Latinos/Hispanics	0.021695	0.016638	0.028644

Table S7. Absolute impacts of the predictors on the final prediction (ordered according to the offline prediction).

5. Regression results

A. Spatial autoregressive lag models. We model the relationship between partisan exposure and voting patterns of all the counties of the contiguous United States with spatial autoregressive lag models (5). We compute spatial weights using k-nearest neighbour, with k equal to 5, 7, and 10. The variables are standardized. The results are the following.

Table S8. Spatial autoregressive lag model with k=5
Relationship between partisan exposure and voting patterns of US counties

	Share of Republican votes			Share of Democratic votes		
	Offline	Online	Residential	Offline	Online	Residential
ρ	-0.204^{***}	-0.030	0.464^{***}	-0.201^{***}	-0.038^{*}	0.562^{***}
	(0.007)	(0.016)	(0.013)	(0.007)	(0.016)	(0.013)
Intercept	0.000	0.000	0.000	0.000	0.000	0.000
	(0.003)	(0.007)	(0.008)	(0.003)	(0.007)	(0.009)
PE_{rep} Offline	1.121^{***}					
	(0.006)					
PE_{rep} Online		0.945^{***}				
		(0.014)				
PE_{rep} Residential			0.598^{***}			
			(0.012)			
PE_{dem} Offline				1.118^{***}		
				(0.006)		
PE_{dem} Online					0.956^{***}	
					(0.013)	
PE_{dem} Residential						0.500^{***}
						(0.013)
R^2	0.971	0.854	0.803	0.967	0.866	0.754
$Log - Likelihood$	1085.806	-1414.123	-1947.910	891.834	-1284.510	-2333.192
AIC	-2163.612	2836.247	3903.820	-1775.667	2577.019	4674.384
N	3098	3098	3098	3098	3098	3098

Note: $^{*}\, p < .05$; $^{**}\, p < .01$; $^{***}\, p < .001$

Table S9. Spatial autoregressive lag model with k=7
Relationship between partisan exposure and voting patterns of US counties

	Share of Republican votes			Share of Democratic votes		
	Offline	Online	Residential	Offline	Online	Residential
ρ	-0.216^{***}	-0.051^{**}	0.485^{***}	-0.211^{***}	-0.055^{***}	0.588^{***}
	(0.007)	(0.017)	(0.013)	(0.008)	(0.016)	(0.013)
Intercept	-0.001	0.000	0.002	0.001	0.000	-0.002
	(0.003)	(0.007)	(0.008)	(0.003)	(0.007)	(0.009)
PE_{rep} Offline	1.125^{***}					
	(0.006)					
PE_{rep} Online		0.959^{***}				
		(0.014)				
PE_{rep} Residential			0.595^{***}			
			(0.012)			
PE_{dem} Offline				1.120^{***}		
				(0.006)		
PE_{dem} Online					0.967^{***}	
					(0.013)	
PE_{dem} Residential						0.496^{***}
						(0.013)
R^2	0.972	0.854	0.803	0.968	0.866	0.754
$Log - Likelihood$	1108.953	-1410.881	-1933.418	904.876	-1281.338	-2312.850
AIC	-2209.905	2829.762	3874.836	-1801.752	2570.677	4633.700
N	3098	3098	3098	3098	3098	3098

Note: $^{*}p < .05$; $^{**}p < .01$; $^{***}p < .001$

Table S10. Spatial autoregressive lag model with k=10
Relationship between partisan exposure and voting patterns of US counties

	Share of Republican votes			Share of Democratic votes		
	Offline	Online	Residential	Offline	Online	Residential
ρ	-0.230^{***}	-0.090^{***}	0.504^{***}	-0.228^{***}	-0.088^{*}	0.613^{***}
	(0.007)	(0.017)	(0.013)	(0.008)	(0.017)	(0.013)
Intercept	-0.001	0.000	0.003	0.001	0.000	-0.003
	(0.003)	(0.007)	(0.008)	(0.003)	(0.007)	(0.009)
PE_{rep} Offline	1.129^{***}					
	(0.006)					
PE_{rep} Online		0.983^{***}				
		(0.014)				
PE_{rep} Residential			0.597^{***}			
			(0.011)			
PE_{dem} Offline				1.126^{***}		
				(0.006)		
PE_{dem} Online					0.988^{***}	
					(0.013)	
PE_{dem} Residential						0.498^{***}
						(0.013)
R^2	0.972	0.855	0.803	0.968	0.867	0.752
$Log - Likelihood$	1145.997	-1401.403	-1926.848	945.742	-1272.408	-2307.772
AIC	-2283.994	2810.805	3861.696	-1883.484	2552.815	4623.545
N	3098	3098	3098	3098	3098	3098

Note: $^{*}p < .05$; $^{**}p < .01$; $^{***}p < .001$

B. Direct, indirect, and total effects in spatial lag models. We compute the direct, indirect, and total effects of the independent variables for spatial autoregressive lag models with $k = 7$. Table S11 outlines the decomposition of these effects.

Table S11. Direct, indirect, and total effects of the independent variables, with standard errors and statistical significance.

Exposure	Vote	Direct	Indirect	Total
PE_{rep}(offline)	Republican	1.131*** (0.006)	-0.206*** (0.007)	0.925*** (0.003)
PE_{rep}(online)	Republican	0.959*** (0.014)	-0.047** (0.015)	0.912*** (0.008)
PE_{rep}(residential)	Republican	0.619*** (0.011)	0.536*** (0.022)	1.155*** (0.021)
PE_{dem}(offline)	Democrat	1.126*** (0.006)	-0.201*** (0.007)	0.925*** (0.003)
PE_{dem}(online)	Democrat	0.968*** (0.013)	-0.051*** (0.015)	0.917*** (0.007)
PE_{dem}(residential)	Democrat	0.530*** (0.012)	0.676*** (0.027)	1.206*** (0.028)

C. OLS models for metro and non-metro areas. We analyse the relationship between partisan exposure and voting patterns for both metropolitan and non-metro areas employing Ordinary Least Squares (OLS) regressions. The variables are standardized. The complete results are the following.

Table S12. OLS models for metropolitan areas
Relationship between partisan exposure and voting patterns

	Share of Republican votes			Share of Democratic votes		
	Offline	Online	Residential	Offline	Online	Residential
Intercept	0.000<br>(0.008)	0.000<br>(0.015)	0.000<br>(0.013)	0.000<br>(0.009)	0.000<br>(0.014)	0.000<br>(0.016)
PE_{rep} Offline	0.963^{***}<br>(0.008)					
PE_{rep} Online		0.853^{***}<br>(0.015)				
PE_{rep} Residential			0.894^{***}<br>(0.013)			
PE_{dem} Offline				0.957^{***}<br>(0.009)		
PE_{dem} Online					0.873^{***}<br>(0.014)	
PE_{dem} Residential						0.833^{***}<br>(0.018)
R^2	0.927	0.727	0.800	0.916	0.763	0.693
N	1156	1156	1156	1156	1156	1156

Note: $^{*}\,p < .05$; $^{**}\,p < .01$; $^{***}\,p < .001$

Table S13. OLS models for non-metro areas
Relationship between partisan exposure and voting patterns

	Share of Republican votes			Share of Democratic votes		
	Offline	Online	Residential	Offline	Online	Residential
Intercept	0.000	0.000	0.000	0.000	0.000	0.000
	(0.003)	(0.006)	(0.014)	(0.003)	(0.005)	(0.016)
PE_{rep} Offline	0.991^{***}					
	(0.003)					
PE_{rep} Online		0.969^{***}				
		(0.006)				
PE_{rep} Residential			0.790^{***}			
			(0.014)			
PE_{dem} Offline				0.993^{***}		
				(0.003)		
PE_{dem} Online					0.973^{***}	
					(0.005)	
PE_{dem} Residential						0.705^{***}
						(0.016)
R^2	0.983	0.939	0.623	0.985	0.947	0.497
N	1942	1942	1942	1942	1942	1942

Note: $^{*}p < .05$; $^{**}p < .01$; $^{***}p < .001$

D. Marginal effects of spatial and OLS models. Fig. S3 shows the total effects of the spatial autoregressive lag models, using spatial weights with $k = 7$, and the marginal effects of the OLS regressions for metropolitan and non-metropolitan areas. All variables have been standardized, so the coefficients can be interpreted as the change in the dependent variable associated with a one-standard-deviation increase in the independent variable.

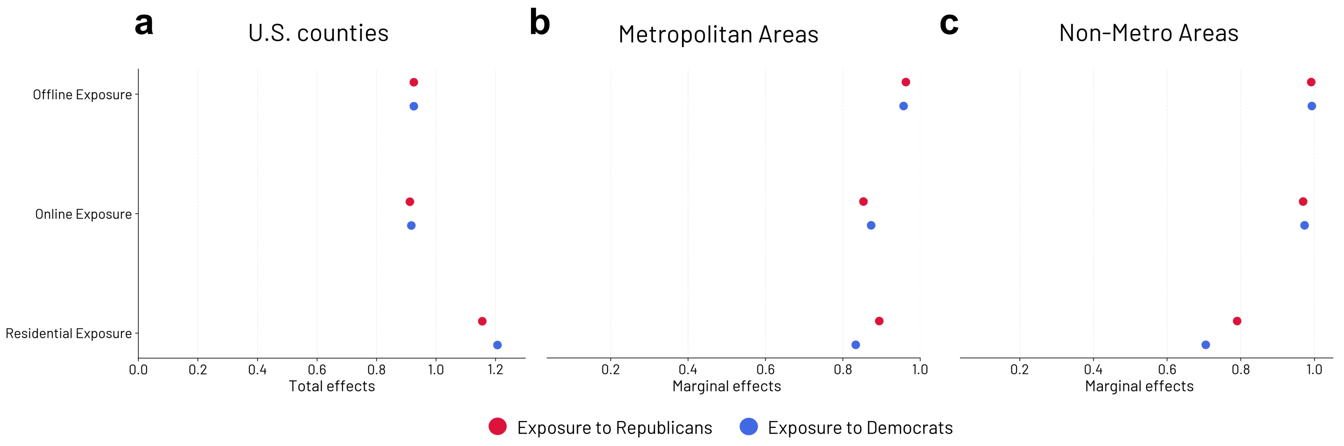

Fig. S3. Total and marginal effects of spatial and OLS models.

6. Dominance analysis results

We enhance the robustness of the results by employing dominance analysis. The results are the following.

Variable	Interactional Dominance	Individual Dominance	Average Partial Dominance	Total Dominance	Percentage Relative Importance
Physical exposure	0.052839	0.962836	0.287563	0.342631	35.287168
Online exposure	0.00135	0.853889	0.212643	0.266387	27.434839
Residential exposure	0.002551	0.697881	0.143587	0.195244	20.107934
% graduated	0.00048	0.209583	0.040195	0.056404	5.808968
% urban population	0.000277	0.213559	0.026400	0.04653	4.792054
% African Americans	0.000844	0.176048	0.027653	0.042851	4.413207
% unemployed	0.000015	0.063053	0.010888	0.016049	1.652886
% Latinos/Hispanics	0.000033	0.017231	0.003634	0.004883	0.502944

Table S14. Dominance Analysis of the relationship between partisan exposure to Republicans (and demographic and socioeconomic characteristics) and votes for the Republican party.

Variable	Interactional Dominance	Individual Dominance	Average Partial Dominance	Total Dominance	Percentage Relative Importance
Physical exposure	0.044584	0.959156	0.299083	0.34978	36.120709
Online exposure	0.001604	0.865488	0.236315	0.285623	29.495423
Residential exposure	0.001567	0.570881	0.110852	0.154695	15.974911
% graduated	0.000479	0.182337	0.040945	0.05356	5.531018
% African Americans	0.000795	0.217876	0.033931	0.052782	5.450677
% urban population	0.000917	0.200255	0.031387	0.048687	5.027732
% unemployed	0.000012	0.074862	0.012022	0.018375	1.897572
% Latinos/Hispanics	0.000011	0.017489	0.003564	0.004861	0.501957

Table S15. Dominance Analysis of the relationship between partisan exposure to Democrats (and demographic and socioeconomic characteristics) and votes for the Democratic party.

Variable	Interactional Dominance	Individual Dominance	Average Partial Dominance	Total Dominance	Percentage Relative Importance
Physical exposure	0.052123	0.927305	0.251718	0.311217	32.578675
Residential exposure	0.011786	0.799454	0.174116	0.231992	24.285288
Online exposure	0.001519	0.726874	0.146281	0.20076	21.015822
% graduated	0.000827	0.297376	0.049967	0.074751	7.82503
% urban population	0.001369	0.326641	0.039834	0.070877	7.419502
% African Americans	0.00166	0.145012	0.022990	0.035577	3.72424
% unemployed	0.000039	0.060931	0.010337	0.015374	1.609367
% Latinos/Hispanics	0.0	0.060495	0.009559	0.014731	1.542076

Table S16. Dominance Analysis of the relationship between partisan exposure to Republicans (and demographic and socioeconomic characteristics) and votes for the Republican party in metropolitan areas.

Variable	Interactional Dominance	Individual Dominance	Average Partial Dominance	Total Dominance	Percentage Relative Importance
Physical exposure	0.051659	0.916374	0.254189	0.311646	32.914846
Online exposure	0.002985	0.762566	0.165927	0.220139	23.250229
Residential exposure	0.007668	0.693444	0.141521	0.19378	20.466278
% graduated	0.00087	0.270723	0.051282	0.072411	7.647734
% urban population	0.003701	0.31066	0.043836	0.072172	7.622512
% African Americans	0.001767	0.180305	0.028092	0.043828	4.628951
% unemployed	0.000067	0.073395	0.011662	0.01793	1.893645
% Latinos/Hispanics	0.000032	0.060556	0.009795	0.01492	1.575805

Table S17. Dominance Analysis of the relationship between partisan exposure to Democrats (and demographic and socioeconomic characteristics) and votes for the Democratic party in metropolitan areas.

A. Dominance analysis results for metro and non-metro areas.

Variable	Interactional Dominance	Individual Dominance	Average Partial Dominance	Total Dominance	Percentage Relative Importance
Physical exposure	0.033474	0.982865	0.322241	0.368723	37.394926
Online exposure	0.001226	0.938518	0.284023	0.330485	33.516907
Residential exposure	0.000508	0.623425	0.140635	0.183468	18.606826
% African Americans	0.000238	0.181181	0.031178	0.046061	4.671355
% graduated	0.000274	0.060213	0.021464	0.023659	2.399414
% unemployed	0.000004	0.084427	0.012937	0.020257	2.054377
% urban population	0.000158	0.049613	0.007214	0.011632	1.179686
% Latinos/Hispanics	0.00009	0.003426	0.001735	0.00174	0.17651

Table S18. Dominance Analysis of the relationship between partisan exposure to Republicans (and demographic and socioeconomic characteristics) and votes for the Republican party in non-metro areas.

Variable	Interactional Dominance	Individual Dominance	Average Partial Dominance	Total Dominance	Percentage Relative Importance
Physical exposure	0.027059	0.985177	0.340459	0.381874	38.695702
Online exposure	0.000725	0.9471	0.308820	0.350093	35.475296
Residential exposure	0.000244	0.497242	0.104400	0.140486	14.235613
% African Americans	0.00012	0.229318	0.039203	0.058082	5.885506
% unemployed	0.000009	0.09863	0.014312	0.023064	2.337096
% graduated	0.000156	0.040722	0.019669	0.019862	2.012615
% urban population	0.000066	0.042312	0.008817	0.01191	1.206865
% Latinos/Hispanics	0.000002	0.003612	0.001389	0.001493	0.151307

Table S19. Dominance Analysis of the relationship between partisan exposure to Democrats (and demographic and socioeconomic characteristics) and votes for the Democratic party in non-metro areas.

7. Random Forest and Elastic Net models

We enhance the robustness and generalizability of the results by training Random Forest and Elastic Net (6) models with k-fold cross-validation ($k = 5$), splitting the dataset into train and test sets with a 70/30 ratio. This analysis is performed considering all the counties of the contiguous United States ($N = 3098$), for both Democrats and Republicans.

Physical partisan exposure is the most important predictor in the Random Forest models, as shown in Table S20. The Elastic Net models achieve the best performances ($R^2 = 0.97$) with $\alpha = 1e-05$ and a ratio between L1 and L2 penalties of 0.1, indicating that it is closer to an L2 (Ridge) penalty in both models. Table S21 shows the results of the beta coefficients.

Variable	Model 1 - Republicans	Model 2 - Democrats
Physical exposure	0.9635	0.4731
Online exposure	0.0033	0.2805
Residential exposure	0.0176	0.1516
% African Americans	0.0035	0.0208
% graduated	0.0032	0.0259
% urban population	0.0058	0.0379
% unemployed	0.0014	0.0056
% Latinos/Hispanics	0.0017	0.0049

Table S20. Variable importance of the Random Forest models.

Variable	Model 1 - Republicans	Model 2 - Democrats
Physical exposure	1.0819	1.1578
Online exposure	-0.1802	-0.2185
Residential exposure	0.0906	0.0625
% African Americans	-0.0310	0.0322
% graduated	-0.0539	0.0536
% urban population	-0.0100	0.0189
% unemployed	0.0117	-0.0080
% Latinos/Hispanics	-0.0058	-0.0027

Table S21. Beta coefficients of the Elastic Net models.

8. Comparison between Colocation Maps and ACS Commuting Flows

We compare the dataset used as a proxy for offline connectedness between US counties (Colocation Maps) with official statistics from the 2016-2020 5-Year ACS Commuting Flows dataset provided by the US Census Bureau. To this aim, we perform the same analyses employed to compare offline, online, and residential exposures.

Similar to the computation of co-location and friendship probabilities, we compute the commuting probability between two counties as the total flows between them divided by their population sizes. We then compute the partisan exposure as captured by the commuting flows and employ both spatial lag model and dominance analysis. We also perform t-tests to compare the difference between offline and commuting partisan exposures.

While the county-level distributions of partisan exposure to Democrats do not show significant differences (Table S22), t-test and Kolmogorov-Smirnov tests reveal significant differences between offline and commuting exposure to Republicans ($P < .001$). Regarding the relationship between partisan exposure and voting patterns in US counties, partisan exposure as captured by commuting flows has slightly lower performance than offline exposure in explaining the variance of voting patterns, both in spatial models and dominance analysis.

Exposure to	Dimension 1	Dimension 2	T-statistic	Significance	KS statistic	Significance
DEM	Offline	Commuting	-0.699	ns	0.214	ns
REP	Offline	Commuting	-3.387	***	0.054	***

Note: $^{*}\, p < .05$; $^{**}\, p < .01$; $^{***}\, p < .001$

Table S22. Comparison between partisan exposure in offline and commuting networks using t-test for the statistical significance.

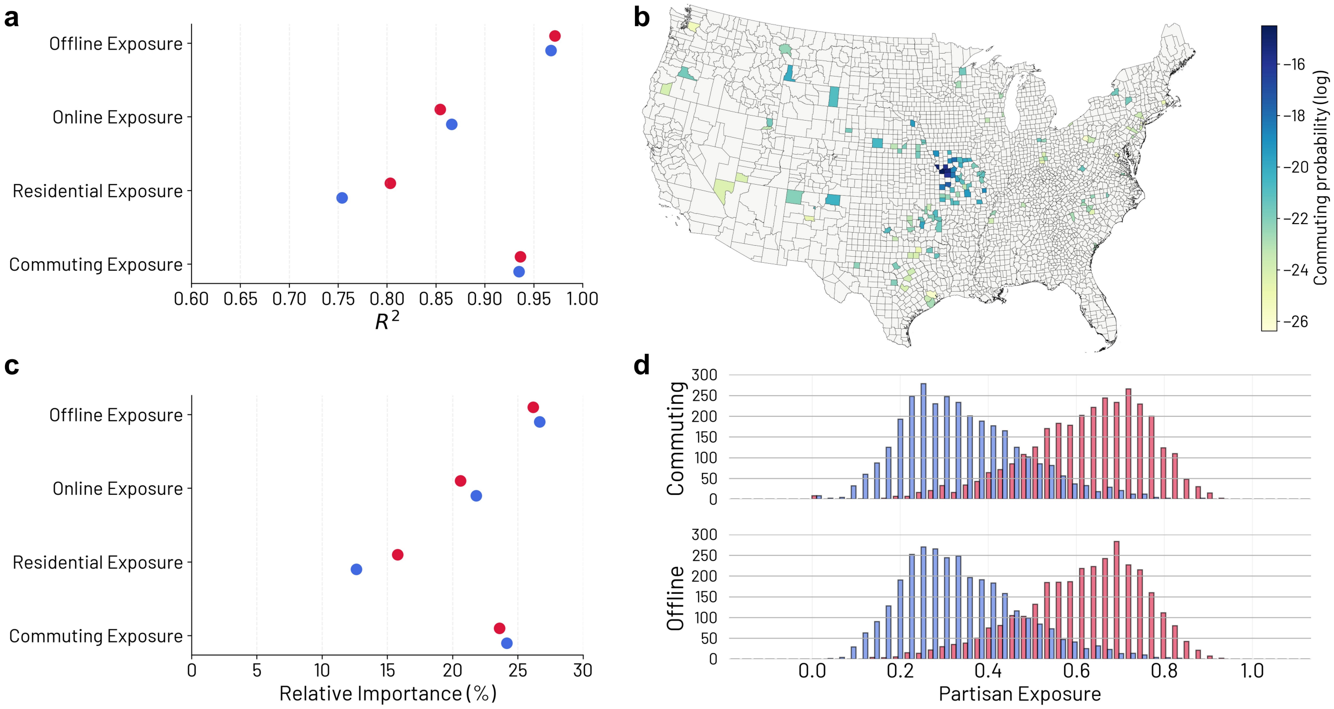

Fig. S4. Comparison between Colocation Maps and ACS Commuting Flows (**a**) R^2 of the spatial lag models. Commuting partisan exposure has a slightly lower R^2 than the offline one. (**b**) Map of the commuting probabilities (log) for the Jackson county. (**c**) Dominance analysis consider all the four dimensions. Commuting partisan exposure shows the lowest relative importance. (**d**) Distributions of offline and commuting partisan exposures to both Democrats and Republicans.

Table S23. Spatial autoregressive lag model with k=7
Relationship between partisan exposure and voting patterns of US counties

	Share of Republican votes		Share of Democratic votes	
	Offline	Commuting	Offline	Commuting
ρ	-0.216^{***}	-0.153^{***}	-0.211^{***}	-0.151^{***}
	(0.007)	(0.011)	(0.008)	(0.011)
Intercept	-0.001	-1.663^{***}	0.001	-0.03
	(0.003)	(0.363)	(0.003)	(0.005)
PE_{rep} Offline	1.125^{***}			
	(0.006)			
PE_{rep} Commuting		1.066^{***}		
		(0.008)		
PE_{dem} Offline			1.254^{***}	
			(0.007)	
PE_{dem} Commuting				1.064^{***}
				(0.009)
R^2	0.972	0.937	0.968	0.935
$Log - Likelihood$	1108.953	-125.143	904.876	-164.699
AIC	-2209.905	258.286	-1801.752	337.398
N	3098	3090	3098	3090

Note: $^{*}\, p < .05$; $^{**}\, p < .01$; $^{***}\, p < .001$

Variable	Interactional Dominance	Individual Dominance	Average Partial Dominance	Total Dominance	Percentage Relative Importance
Physical exposure	0.020545	0.962702	0.186332	0.254175	26.17196
Commuting exposure	0.0003	0.931946	0.161400	0.229117	23.591774
Online exposure	0.001613	0.853406	0.135055	0.200045	20.598325
Residential exposure	0.002468	0.697649	0.097064	0.153285	15.783504
% graduated	0.000462	0.20697	0.027305	0.044285	4.559962
% urban population	0.000334	0.211635	0.019205	0.03849	3.963212
% African Americans	0.000862	0.177088	0.019570	0.034993	3.603222
% unemployed	0.000014	0.063017	0.007591	0.012907	1.329055
% Latinos/Hispanics	0.000029	0.01708	0.002538	0.003875	0.398987

Table S24. Dominance Analysis of the relationship between partisan exposure to Republicans (and demographic and socioeconomic characteristics) and votes for the Republican party by including commuting exposure.

Variable	Interactional Dominance	Individual Dominance	Average Partial Dominance	Total Dominance	Percentage Relative Importance
Physical exposure	0.020344	0.95901	0.192265	0.258356	26.67709
Commuting exposure	0.000183	0.930383	0.167888	0.233976	24.159666
Online exposure	0.001774	0.864938	0.147705	0.211183	21.806092
Residential exposure	0.001593	0.570121	0.075455	0.122211	12.61917
% African Americans	0.000834	0.219189	0.023897	0.043034	4.443513
% graduated	0.000482	0.179621	0.027598	0.041477	4.282771
% urban population	0.000856	0.198261	0.022310	0.039476	4.076222
% unemployed	0.000012	0.074858	0.008384	0.01484	1.532337
% Latinos/Hispanics	0.000005	0.017331	0.002543	0.003904	0.40314

Table S25. Dominance Analysis of the relationship between partisan exposure to Democrats (and demographic and socioeconomic characteristics) and votes for the Democratic party by including commuting exposure.

9. Exclude local exposure (self-loops) in the networks

To achieve a comprehensive understanding of the relationship between partisan exposure and voting patterns, we perform all the analyses outlined in the paper excluding the self-loops from the co-location and online friendship networks. A self-loop refers to either the co-location or friendship probability between a county i and itself. In the physical network, local exposure represents a substantial and fundamental aspect of an individual's exposure. Conversely, in the online network, while local exposure still has a huge impact, online exposure tends to be more heterogeneous and characterized by greater external exposure.

Excluding local exposure in the networks, physical proximity shows a significant loss of predictive and explanation power in determining US political outcomes at the county level (Fig. S5). This is confirmed by both the spatial model (Fig. S5a) and dominance analysis (Fig. S5d) and both metropolitan (Fig. S5b and S5e) and non-metropolitan areas (Fig. S5c and S5f). Thus, online partisan exposure outweighs offline proximity in predicting US counties voting patterns, with greater differences between online and offline partisan exposure to Democrats.

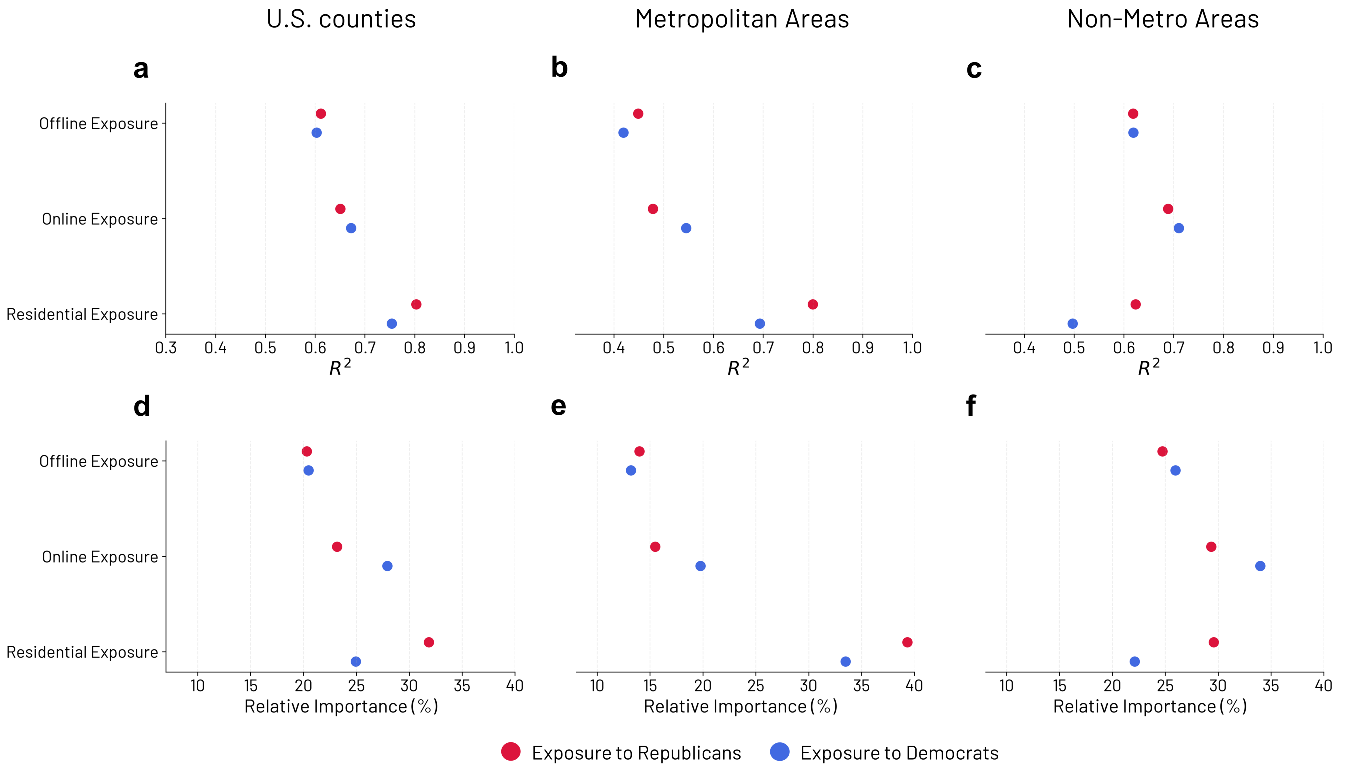

Fig. S5. Relative contribution of the three dimensions of partisan exposure on voting patterns, excluding local exposure in the networks. (**a**) R^2 of the spatial models with $k = 7$ for all the counties in the contiguous United States. (**b**) R^2 of the OLS models for the metropolitan areas (RUCC 1–3). (**c**) R^2 of the OLS models for the non-metropolitan areas (RUCC 4–9). (**d**) Dominance analysis for all the US counties, considering the three dimensions, along with demographic and socioeconomic controls. (**e**) Dominance analysis for the metropolitan areas (RUCC 1–3). (**f**) Dominance analysis for the non-metropolitan areas (RUCC 4–9).

10. Descriptive and regression analyses of survey data

We leverage the 2020-2022 Social Media Study provided by the American National Election Studies (ANES) to investigate the relationship between partisan exposure and voting behavior at the individual level. As explained in the Materials and Methods Section, we consider only those respondents who declared their vote preference in the second wave, have valid voting records, and responded to all the questions of interest. The final sample consists of 2,420 respondents, characterized as follows.

Table S26. Descriptive statistics of the analytical sample ($N = 2420$)

Variable	Mean	SD	Min	Max
Gender	1.50	0.50	1	2
Age	50.59	15.88	18	80
Education - Graduation (dummy)	0.45	0.50	0	1
Ethnicity: White	0.71	0.45	0	1
Ethnicity: Black	0.10	0.31	0	1
Ethnicity: Hispanic	0.11	0.32	0	1
Employment	2.67	2.14	1	7
Income	10.60	4.02	1	18
W1 - Offline Exposure to Democrats	3.08	1.08	1	5
W1 - Online Exposure to Democrats	3.11	0.99	1	5
W1 - Offline Exposure to Republicans	2.83	1.04	1	5
W1 - Online Exposure to Republicans	2.76	0.94	1	5
W2 - Offline Exposure to Democrats	3.08	1.07	1	5
W1 - Online Exposure to Democrats	3.05	1.06	1	5
W2 - Offline Exposure to Republicans	2.85	1.04	1	5
W2 - Online Exposure to Republicans	2.74	1.01	1	5
Political vote (0 Democrat - 1 Republican)	0.42	0.49	0	1

Table S27. Logit models –
Relationship between partisan exposure and political vote (0 Democrat - 1 Republican)

	Exposure to Democrats		Exposure to Republicans	
	Wave 1	Wave 2	Wave 1	Wave 2
Intercept	3.553^{***}	3.210^{***}	-3.518^{***}	-3.923^{***}
	(0.260)	(0.245)	(0.254)	(0.263)
$PE_{offline}$	-0.976^{***}	-0.935^{***}	0.707^{***}	1.029^{***}
	(0.066)	(0.066)	(0.062)	(0.071)
PE_{online}	-0.366^{***}	-0.318^{***}	0.493^{***}	0.266^{***}
	(0.065)	(0.062)	(0.066)	(0.066)
Metro area	-0.357^{**}	-0.330^{*}	-0.460^{***}	-0.381^{**}
	(0.136)	(0.133)	(0.132)	(0.134)
Ethnicity: White	0.626^{***}	0.722^{***}	0.426^{**}	0.446^{**}
	(0.147)	(0.145)	(0.142)	(0.147)
Ethnicity: Black	-1.202^{***}	-1.334^{***}	-1.455^{***}	-1.303^{***}
	(0.287)	(0.293)	(0.282)	(0.285)
Ethnicity: Hispanic	0.453^{*}	0.530^{**}	0.535^{**}	0.626^{**}
	(0.194)	(0.192)	(0.184)	(0.192)
Education	-0.194	-0.234^{*}	-0.393^{***}	-0.426^{***}
	(0.110)	(0.110)	(0.106)	(0.109)
AIC	2942.8	2969.1	3110.5	2981.8
N	2420	2420	2420	2420

Note: $^{*}p < .05$; $^{**}p < .01$; $^{***}p < .001$

References

1. Gordon Willard Allport, Kenneth Clark, and Thomas Pettigrew. The nature of prejudice. 1954.
2. Ryan D Enos. Causal effect of intergroup contact on exclusionary attitudes. *Proceedings of the National Academy of Sciences*, 111(10):3699–3704, 2014.
3. Ryan D Enos. *The space between us: Social geography and politics*. Cambridge University Press, 2017.
4. Noah L Nathan and Melissa L Sands. Context and contact: Unifying the study of environmental effects on politics. *Annual Review of Political Science*, 26(1):233–252, 2023.
5. L. Anselin. *Spatial Econometrics: Methods and Models*. Kluwer Academic Publishers, Dordrecht, 1988.
6. Hui Zou and Trevor Hastie. Regularization and variable selection via the elastic net. *Journal of the Royal Statistical Society Series B: Statistical Methodology*, 67(2):301–320, 2005.
